# Noninterventional follow‐up vs fluid bolus in RESPONSE to oliguria—The RESPONSE trial protocol and statistical analysis plan

**DOI:** 10.1111/aas.13599

**Published:** 2020-04-28

**Authors:** Nina Inkinen, Tuomas Selander, Ville Pettilä, Miia Valkonen, Minna Bäcklund, Johanna Wennervirta, Anni Pulkkinen, Johanna Hästbacka, Suvi T. Vaara

**Affiliations:** ^1^ Department of Anesthesia and Intensive Care Central Finland Central Hospital Central Finland Health Care District Jyväskylä Finland; ^2^ Division of Intensive Care Medicine Department of Anesthesiology, Intensive Care and Pain Medicine University of Helsinki and Helsinki University Hospital Helsinki Finland; ^3^ Science Service Center Kuopio University Hospital Kuopio Finland

**Keywords:** acute kidney injury, critically ill, fluid bolus, oliguria

## Abstract

**Background:**

Oliguria is a frequent trigger for administering a fluid bolus, but the effect of fluid bolus in improving urine output is inadequately demonstrated. Here, we summarize the protocol and detailed statistical analysis plan of the randomized, controlled RESPONSE trial comparing follow‐up as the experimental group and a 500 mL crystalloid fluid bolus as the control group for oliguria in critically ill oliguric patients.

**Methods:**

Our trial is an investigator‐initiated, randomized, controlled, pilot trial conducted in three ICUs in two centers. We aim to randomize 1:1 altogether 130 hemodynamically stable oliguric patients either to a 2‐hour follow‐up without interventions or to receive a crystalloid bolus of 500 mL over 30 minutes. The primary outcome is the change in individual urine output during the 2‐hour period compared to 2 hours preceding randomization. Doubling of the urine output is considered clinically significant. Additionally, we record the duration of oliguria, physiological and biochemical variables, adverse events, and the incidences of acute kidney injury and renal replacement therapy.

**Conclusions:**

Oliguria is a frequent trigger for potentially harmful fluid loading. Therefore, the RESPONSE trial will give information of the potential effect of fluid bolus on oliguria in critically ill patients.

**Trial registration:**

clinical.trials.gov, NCT02860572.

## 1 INTRODUCTION

Fluid bolus therapy is a widely used intervention in the treatment of critically ill patients.^1^, ^2^ Fluid boluses are typically administered in the optimization phase of fluid resuscitation aiming to optimize and maintain adequate organ perfusion by increasing stroke volume.^3^ After hypotension, oliguria (urine output less than 0.5 mL/kg/h) was the most common trigger to administer a fluid bolus.^2^ Albeit oliguria is often seen as a manifestation of inadequate organ perfusion that can be due to hypovolemia, distributive shock, or depressed cardiac function, it can also be a manifestation of early acute kidney injury (AKI).^4^

Regrettably, the response to fluid bolus is currently poorly documented in clinical practice.^2^ A recent systematic review among septic patients found no previous randomized trials comparing the efficacy of fluid bolus therapy to alternative interventions.^1^ Moreover, few observational studies have reported data regarding urine output in response to fluid bolus therapy, and these data suggest no clinically meaningful response.^1^ A retrospective analysis among patients with acute respiratory distress syndrome in a randomized trial comparing conservative vs liberal fluid management found that urine output did not significantly increase within 4 hours after a fluid bolus.^5^ A small observational study among septic shock patients did not find fluid boluses to increase urine output^6^ whereas another study reported urine output to increase approximately by 50% both among patients with oliguria and AKI after a large fluid bolus (median volume 1224 mL crystalloid or colloid).^7^ Additionally, improvement in systemic hemodynamics after fluid bolus has been found to correlate poorly with renal hemodynamics,^7^, ^8^, ^9^, ^10^ further complicating the assessment of potential benefits of fluid bolus in terms of reversing oliguria. Moreover, the effect of fluid bolus on cardiovascular variables is short‐lived among patients in shock^11^, ^12^ suggesting that fluid boluses in the optimization phase of shock are unlikely to improve patient‐centered outcomes.

A typically administered fluid bolus is 500 mL of crystalloid given over 30 minutes,^2^ and fluid boluses may constitute a marked proportion, up to 50%, of the fluid amount administered daily.^6^ A growing body of evidence suggests a poor renal response to fluid bolus,^5^, ^6^ which particularly among oliguric patients translates to an increased risk of fluid accumulation. Moreover, a growing body of evidence shows association of fluid accumulation with increased risk of mortality.^13^, ^14^, ^15^, ^16^

Therefore, in oliguric critically ill patients, clinical equipoise exists in conducting a randomized trial that aims to compare a follow‐up approach without any fluid bolus to standard therapy with fluid bolus (500 mL of crystalloid administered over 30 minutes). We hypothesize that the incidence of doubling of urine output will not differ between the groups to a clinically significant level defined as at least a 20% absolute increase in the number of responders.

## 2 METHODS

### 2.1 Trial design and setting

The RESPONSE trial is an investigator‐initiated, open, randomized, controlled, pilot study conducted in two Finnish centers; two intensive care units (ICUs) in Helsinki University Hospital (Helsinki) and in the ICU of Central Finland Central Hospital (Jyväskylä).

### 2.2 Trial registration

The RESPONSE trial was registered in the clinicaltrials.gov registry (identifier NCT02860572) on 9 August 2016.

### 2.3 Trial conduct

The study protocol has been prepared in accordance with the Standard Protocol Items: Recommendations for Interventional Trials (SPIRIT) guidelines.^17^ The study will be conducted according to the Declaration of Helsinki and its later amendments and according the Good Clinical Practice guidelines.

### 2.4 Randomization

Eligible patients will be randomized 1:1 within 1 hour of full eligibility using computer‐based algorithm created by an independent statistician and web‐based allocation concealment for randomization. Randomization is stratified according to the presence/absence of AKI (defined by KDIGO criteria^4^) and sepsis (according to the Sepsis definition^18^). Permutated blocks of varying size (4, 6, or 8) are used.

### 2.5 Blinding

Due to the nature of the study, blinding of the ICU personnel is not feasible. The allocation is blinded for the statistician conducting the data analysis. Additionally, we will write an abstract of study results prior to becoming aware of the group allocation and will include this abstract in the appendix of the original publication. Use of randomization blocks of varying size not known for clinicians prevents knowledge of treatment allocation before randomization.

### 2.6 Inclusion and exclusion criteria

All admissions to ICU will be screened. If the initial (static) inclusion or exclusion criteria (marked with asterisk) are not excluding the patient becoming eligible for randomization if oliguria develops, the patient will be followed further. If oliguria develops, the presence of dynamic exclusions will be rechecked, and if none of those is present, patient is eligible for randomization.

#### 2.6.1 Inclusion criteria

- Age over 18*
- Emergency admission to an ICU*
- Mean arterial pressure (MAP) >65 mm Hg (with vasopressors if needed) and initial fluid resuscitation for shock/hypovolemia has been given
- Oliguria (urine output less than 0.5 mL/kg/h) for at least two consecutive hours

#### 2.6.2 Exclusion criteria

- Marked fluctuations in hemodynamics within the last 2 hours (cardiac arrhythmias, increase in norepinephrine dose over 0.2 µg/kg/min, initiation of inotrope/inodilator)
- Administration of furosemide within last 6 hours
- Chronic kidney disease (estimated pre‐critical illness GFR < 60 mL/min/1.73 m^2^)*
- Renal replacement therapy*
- Urgent indications for commencing renal replacement therapy for AKI
- Fluid overload (cumulative fluid accumulation exceeds 10% of baseline body weight)^13^
- Pulmonary edema (bilateral infiltrates in chest x‐ray)
- Active bleeding (need for transfusion, platelets, or fresh frozen plasma)
- Suspected or known intra‐abdominal hypertension (IAP > 16 mm Hg)
- Pregnant or lactating*
- Expected survival less than 24 hours
- Obtaining informed written consent is not possible/ consent is denied

The detailed definitions of inclusion and exclusion criteria are presented in the Supplement.

### 2.7 Trial interventions

The timeline of eligibility, randomization, and study interventions are presented in Table 1. The intervention period lasts 2 hours.

**TABLE 1:**
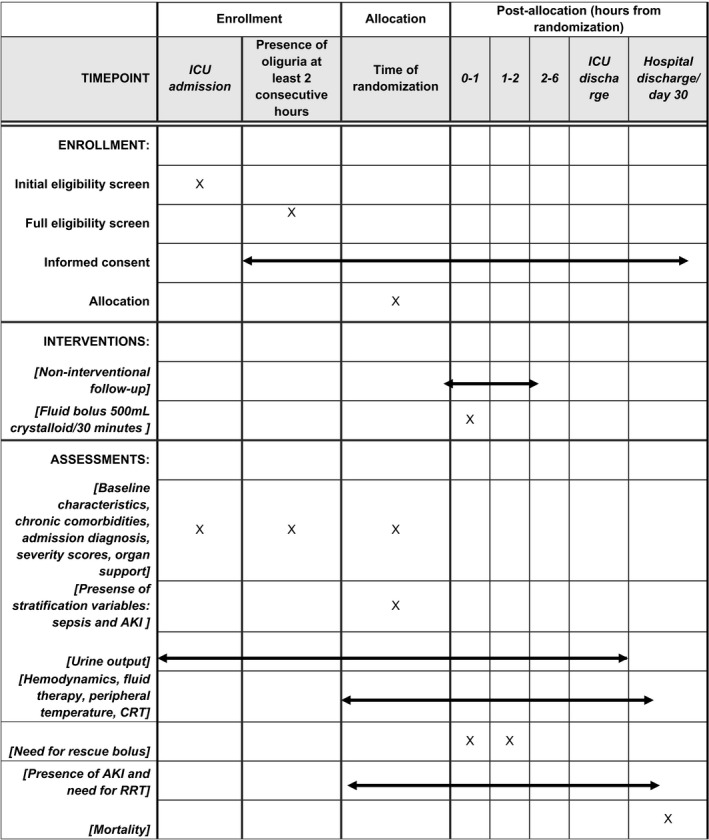
Overview of the schedule of enrollment, interventions, and assessments according to the SPIRIT 2013 statement

Abbreviations: AKI, acute kidney injury; CRT, capillary refill time; RRT, renal replacement therapy.

#### 2.7.1 Intervention group (noninterventional follow‐up)

Patients in the intervention group will not receive fluid bolus or diuretics to increase urine output during the 2‐hour intervention period.

#### 2.7.2 Standard group (fluid bolus group)

Patients will receive 500 mL intravenous crystalloid (Ringer's acetate) infused over 30 minutes using an infusion pump.

#### 2.7.3 Concomitant treatment

In both groups, the infusion rate of all on‐going infusions such as nutrition and maintenance fluids will be kept constant during the 2‐hour period. Vasoactive drugs, short‐acting insulin, sedation, and other medications can be titrated according to the discretion of the clinician. No diuretics during the 2‐hour study period are allowed. Modifying the targeted mean arterial pressure (MAP) and adjusting vasopressor dose accordingly (higher MAP potentially increasing urine output^19^) are not recommended in either of the groups.

If severe hemodynamic instability develops during the 2‐hour study period due to suspected hypovolemia (defined as a need to increase norepinephrine‐infusion >0.2 µg/kg/min from baseline or heart rate increase >30 beats/min from baseline), a rescue bolus of 500 mL crystalloid over 30 minutes in both groups can be administered according to the decision of the treating clinician.

### 2.8 Outcome measures

#### 2.8.1 Primary outcome measure

**Figure 1:**
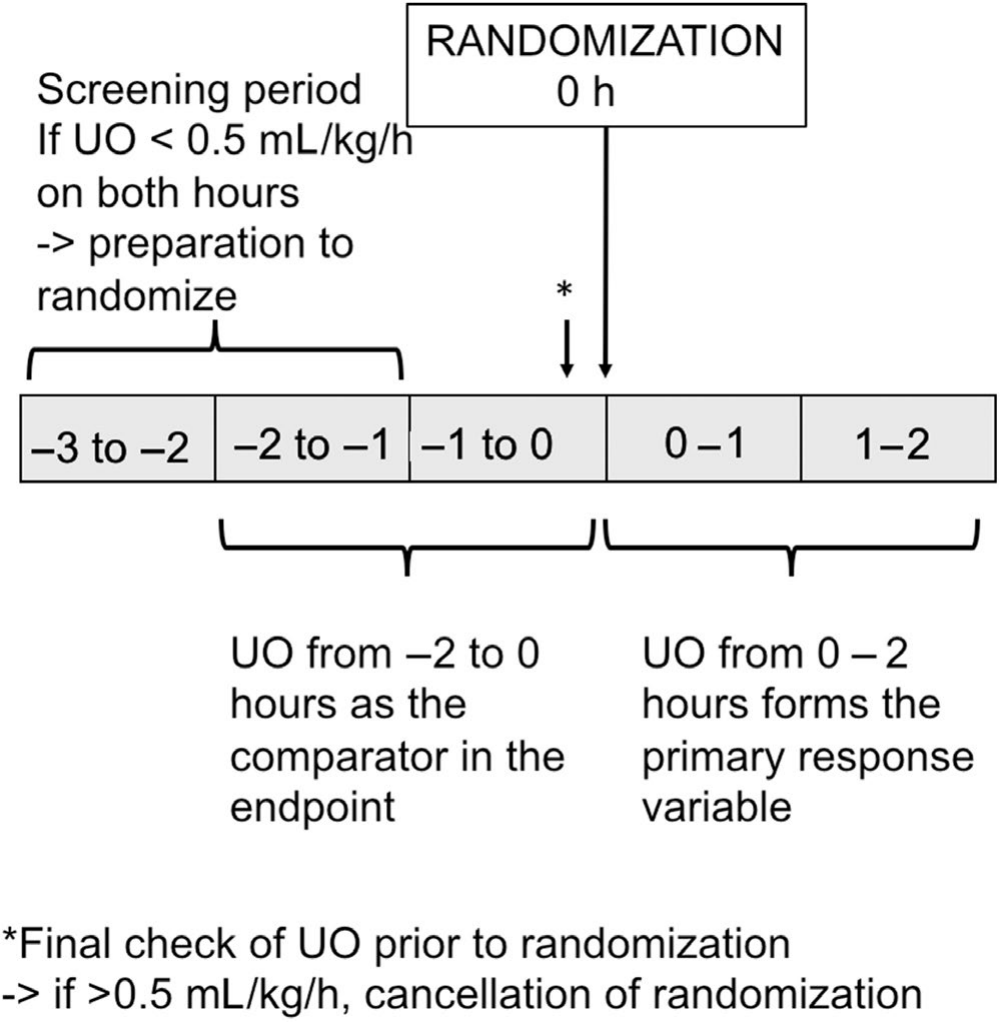
Schematic presentation of determination of urine output for the primary endpoint variable. UO, urine output

The primary outcome is defined the individual mean cumulative 2‐hour urine output (mL/kg/h) 2 hours after randomization divided by the mean cumulative 2‐hour urine output (mL/kg/h) measured 2 hours preceding randomization expressed as a percentage (Figure 1). Based on a previous prospective cohort study, we consider doubling of the urine output as a clinically significant increase in urine output among oliguric patients,^20^ if urine output increases at least 10 mL/h (ie difference between cumulative 2‐hour urine output post‐randomization vs pre‐randomization is at least 20 mL).^21^ The proportion of patients who doubled their urine output in both groups will be compared.

Choosing the 2‐hour timeline for the endpoint was based on assumption that urine output in response to fluid bolus would increase in that time based on previous studies.^22^, ^23^, ^24^ In studies among healthy volunteers, the median/mean time to micturition after a large crystalloid bolus (saline 1 liter of over 30 minutes or 30 mL/kg administered 100 mL/min) has been 82‐112 minutes.^22^, ^23^ Moreover, 2‐hour cumulative urine output in response to Furosemide stress test performed best in predicting worsening of AKI.^24^

#### 2.8.2 Secondary outcome measures

- The difference between groups in the change in individual urine output (with 95% CIs) at 2 hours
- Duration of consecutive oliguria (urine output <0.5 mL/kg)
  - Hours from randomization, assessed until resolved for the first time/patients is discharged/RRT is commenced)
- Cumulative fluid balance on study day
  - 6 hours from randomization

### 2.9 Exploratory outcomes

- Physiological effects from randomization until measured at 2 hours
  - Mean arterial pressure
  - Heart rate
  - Norepinephrine dose
  - Central venous pressure (among those with central line)
  - Difference in core vs peripheral temperature
  - Capillary refill time (until 60 minutes)
  - Blood pH
  - Standard base excess
  - Arterial lactate
- Number of patients receiving rescue boluses and the number of rescue boluses
  - From randomization to 2 hours post‐randomization
- Number of patients with one or several protocol violation(s) and the number of those per patient
  - From randomization to 2 hours post‐randomization

Protocol violations include the following:

- Patient received Furosemide or other diuretic during the 2‐hour period
- Changing MAP target (defined as MAP level increased from baseline target AND increased vasopressor dose from the baseline)
- No full 500 mL bolus in bolus group
- Fluid bolus (other than rescue bolus) in the follow‐up group
- Erroneously randomized non‐eligible patient

d. Number of patients with adverse events
  - Assessed from randomization until the next morning
  - Defined as events reported by the treating clinician in the open questionnaire included in the protocol
e. Highest AKI stage within
  (i) 24 hours
  (ii) 48 hours
  (iii) During ICU stay
f. Number of patients receiving renal replacement therapy (during ICU stay)

### 2.10 Data collection

Trained research personnel will perform data collection using an electronic platform (Absolute Imaginary Software Ltd). Data S1 comprises the detailed list of collected data items. In brief, patient characteristics at baseline will be collected, and details of urine output, hemodynamics, and fluid therapy will be recorded during the intervention and follow‐up period. Patients’ vital status will be recorded at hospital discharge or on day 30 whichever comes first.

### 2.11 Statistical analysis

#### 2.11.1 General analytical principles

Statistical analyses will be performed on the intention‐to‐treat (ITT) population defined as all randomized subject excluding (a) those without consent (b) ineligible subjects who were erroneously randomized and did not receive the trial intervention. The conclusions of the analysis will be based on the ITT analysis.

A sensitivity analysis will be conducted in the per‐protocol population, defined as the ITT population and excluding subjects (a) who experienced one or several protocol violations or (b) who received the rescue bolus.

Continuous variables will be checked for normality using the Shapiro‐Wilk test. We will set statistical significance to .05 and report two‐sided *P*‐values.

#### 2.11.2 Primary outcome

The primary outcome between follow‐up and fluid bolus groups will be adjusted for the two stratification variables^25^ (presence of AKI and presence of sepsis) using two‐tailed logistic regression. Obtained odds ratios (OR) will be converted to risk ratios (RR) with 95% confidence intervals (CI). Additionally, we will report crude event rates and risk ratios.

#### 2.11.3 Secondary and exploratory outcomes

As the primary outcome, the secondary and exploratory outcomes will be adjusted for the two stratification variables.^25^ Continuous measures will be expressed according to their distribution and difference between the groups in the change in individual urine output, duration of consecutive oliguria, and cumulative fluid balance on study day will be compared using mean or median regression depending on normal distribution and expressed with 95% CIs. Dichotomous endpoint measures (number of patients receiving rescue boluses, highest AKI stage within 24, 48 hours and during ICU stay, and number of patients receiving RRT) will be analyzed using ordinal or logistic regression and reported as risk ratios with 95% CIs. Furthermore, crude event rates and risk rations will be reported for dichotomous outcomes and absolute differences with 95% CIs for continuous outcomes.

#### 2.11.4 Other variables

Continuous variables will be compared with Mann‐Whitney *U *test or Student's *t* test depending on normal distribution, and categorical data using Chi‐squared test.

#### 2.11.5 Missing data

We do not expect to have missing data about the primary outcome measure or stratification variables in the ITT population. Regarding secondary or exploratory outcomes, some missingness is expected (for example CVP values in patients who have not a central line inserted), but imputation will not be used. Regarding other data points, if the amount of missing observations regarding other variables is less than 5%, we do not impute data. Regarding descriptive data, an appropriate multiple imputation strategy according to the type of missingness (missing at random or missing completely at random) will be used if missingness exceeds 5% of observations.

### 2.12 Sample size

No reliable previous data to inform about the incidence of the primary outcome were available. Among oliguric (urine output <0.5 mL/kg/h for at least 3 hours), hemodynamically stable patients, 50% were considered as renal responders to a fluid challenge that constituted at least one 500 mL crystalloid bolus administered over 15 minutes.^10^ In that study the definition of a positive renal response (post‐bolus urine output over 0.5 mL/kg/h for at least 3 hours) was more liberal than in our trial.^10^ Fluid bolus did not significantly increase urine output among patients with acute respiratory distress syndrome (both oliguric and non‐oliguric).^5^ Therefore, we assume that 30% of patients in the fluid bolus group will be endpoint‐positive. Additionally, we consider 20% absolute difference (which translates to a number needed to treat of 5) as a minimum clinically meaningful difference between the groups to support the use of such a common and inexpensive intervention. Thus, to reach a 20% absolute difference in the primary outcome (30% in the fluid bolus group and 10% in the noninterventional follow‐up group), 62 patients per group will be required to reach 80% power with significance level set at .05 (two‐sided). Considering possible drop‐outs we aim to randomize 65 patients per group to target at least 62 patients per group in the ITT population.

### 2.13 Pre‐planned subgroup analyses

The primary and secondary outcome measures will be analyzed in two pre‐defined subgroups: (a) patients with sepsis according to sepsis‐3 definition^18^ and (b) patients with AKI defined by the KDIGO criteria.^4^ Presumably, patients with AKI may exert a poorer response in terms of urine output to fluid bolus than those without AKI. Additionally, the recent pathophysiological hypotheses regarding oliguria in sepsis suggest that oliguria may be an adaptive mechanism to severe inflammation,^26^ suggesting also a lower incidence of reversal of oliguria. The results obtained in the subgroups will be interpreted as hypothesis generating and heterogeneity between subgroups will be assessed by *I* ^2^‐test.

### 2.14 Trial Profile

The flow of trial participants will be reported according to the CONSORT statement.^27^

### 2.15 Data monitoring and safety committee

As this is a low‐risk pilot trial, no data and safety monitoring committee (DSMB) will be formed. Informed consents, inclusion and exclusion criteria, and collected data will be monitored.

### 2.16 Interim analyses

No interim analyses will be conducted.

## 3 DISCUSSION

Albeit fluid bolus therapy is frequently used in oliguria to increase urine output, the success of this intervention is largely unknown. The RESPONSE trial will give high‐quality information about the physiological outcomes of fluid bolus therapy given for oliguria in hemodynamically stable patients compared to a follow‐up treatment strategy without additional fluids. Conservative fluid resuscitation strategies especially in septic patients are actively investigated^28^, ^29^ as the evidence regarding harms of fluid overload are being increasingly recognized, especially among patients with AKI.^30^, ^31^ The results of RESPONSE trial will help to assess the potential benefit of fluid bolus therapy, and if no clinically meaningful improvement on urine output or other physiological variables can be demonstrated, will help in avoiding unnecessary fluid loading in the future.

The randomized approach in studying the response of fluid bolus therapy to an alternative intervention is an obvious strength of our study, as the current evidence is based on observational data. Moreover, we are carefully recording data on other fluid input and output, common hemodynamic parameters, acid‐base balance, and blood gases. Thus, we will be able to provide a detailed analysis on the effects of fluid bolus on these parameters as compared to the follow‐up approach. Additionally, we will include a heterogeneous population of critically ill patients with oliguria who have received the initial fluid resuscitation, which will improve the generalizability of our results.

Our trial has some obvious limitations. First, the intervention is not blinded from the clinical team or the investigators due to its nature. Potentially, clinician aware of the patient being in the follow‐up group may give a fluid bolus after the intervention period, and therefore neutralizing the difference between the groups regarding fluid balance. However, we record the details of urine output and fluid therapy until 6 hours post‐randomization to assess the administration additional fluid boluses. Moreover, the trial statistician will be blinded to the group allocation. Second, our trial protocol does not mandate cardiac output monitoring, and therefore, commenting whether patients were hemodynamic fluid responders will not be possible. However, numerous studies have shown that global hemodynamics and renal hemodynamics do not correlate.^7^, ^10^

In conclusion, the RESPONSE trial will bring new evidence about the effects of fluid bolus therapy compared to noninterventional follow‐up on urine output. These results will help to allocate fluid bolus therapy to those patients who benefit from it.

## 4 ETHICAL CONSIDERATIONS AND CONSENT TO PARTICIPATE

The Ethics Committee of Department of Surgery, Hospital district of Helsinki and Uusimaa (HUS/1308/2016), approved the trial. Because of the critically ill patient population and a time‐sensitive standard group intervention, deferred consent was approved with an informed, written consent obtained from the patients’ next of kin as soon as possible and confirmed from the patient if possible. Patient will be asked to provide the consent prior to randomization if his/her clinical situation allows. Written informed consent is mandatory for all patients included in the final analysis.

## 5 DATA SHARING STATEMENT

The de‐identified trial data set will be published as a supplement to the original publication.

## 6 DISSEMINATION

Results of the study will be submitted to a peer‐reviewed medical journal regardless of the results.

## CONFLICT OF INTEREST

The authors report no conflicts of interest.

## Supporting information

Supplementary Material

## References

[1] Glassford NJ , Eastwood GM , Bellomo R . Physiological changes after fluid bolus therapy in sepsis: a systematic review of contemporary data. Crit Care. 2014;18:696. 25673138 10.1186/s13054-014-0696-5 PMC4331149

[2] Cecconi M , Hofer C , Teboul J‐L , et al. Fluid challenges in intensive care: the FENICE study: a global inception cohort study. Intensive Care Med. 2015;41:1529‐1537. 26162676 10.1007/s00134-015-3850-x PMC4550653

[3] Hoste EA , Maitland K , Brudney CS , et al. Four phases of intravenous fluid therapy: a conceptual model. Br J Anaesth. 2014;113:740‐747. 25204700 10.1093/bja/aeu300 PMC6863743

[4] Kidney Disease: Improving Global Outcomes (KDIGO) Acute Kidney Injury Work Group . KDIGO clinical practice guideline for acute kidney injury. Kidney Int. 2012:1‐138.

[5] Lammi MR , Aiello B , Burg GT , et al. Response to fluid boluses in the fluid and catheter treatment trial. Chest. 2015;148(4):919‐926. 26020673 10.1378/chest.15-0445 PMC4694152

[6] Bihari S , Prakash S , Bersten AD . Post resusicitation fluid boluses in severe sepsis or septic shock: prevalence and efficacy (price study). Shock (Augusta, Ga). 2013;40:28‐34. 10.1097/SHK.0b013e31829727f1 23635850

[7] Moussa MD , Scolletta S , Fagnoul D , et al. Effects of fluid administration on renal perfusion in critically ill patients. Crit Care. 2015;19:250. 26070308 10.1186/s13054-015-0963-0 PMC4488122

[8] Prowle JR , Molan MP , Hornsey E , Bellomo R . Measurement of renal blood flow by phase‐contrast magnetic resonance imaging during septic acute kidney injury: a pilot investigation. Crit Care Med. 2012;40:1768‐1776. 22487999 10.1097/CCM.0b013e318246bd85

[9] Prowle JR , Ishikawa K , May CN , Bellomo R . Renal plasma flow and glomerular filtration rate during acute kidney injury in man. Ren Fail. 2010;32:349‐355. 20370451 10.3109/08860221003611695

[10] Legrand M , Le Cam B , Perbet S , et al. Urine sodium concentration to predict fluid responsiveness in oliguric ICU patients: a prospective multicenter observational study. Crit Care. 2016;20:165. 27236480 10.1186/s13054-016-1343-0 PMC4884621

[11] Aya HD , Ster IC , Fletcher N , Grounds RM , Rhodes A , Cecconi M . Pharmacodynamic analysis of a fluid challenge. Crit Care Med. 2016;44:880‐891. 26683506 10.1097/CCM.0000000000001517

[12] Nunes TS , Ladeira RT , Bafi AT , de Azevedo LC , Machado FR , Freitas FG . Duration of hemodynamic effects of crystalloids in patients with circulatory shock after initial resuscitation. Ann Intensive Care. 2014;4:25. 25593742 10.1186/s13613-014-0025-9 PMC4273721

[13] Bouchard J , Soroko SB , Chertow GM , et al. Fluid accumulation, survival and recovery of kidney function in critically ill patients with acute kidney injury. Kidney Int. 2009;76:422‐427. 19436332 10.1038/ki.2009.159

[14] Vaara ST , Korhonen A‐M , Kaukonen K‐M , et al. Fluid overload is associated with an increased risk for 90‐day mortality in critically ill patients with renal replacement therapy: data from the prospective FINNAKI study. Crit Care. 2012;16:R197. 23075459 10.1186/cc11682 PMC3682299

[15] Garzotto F , Ostermann M , Martín‐Langerwerf D , et al. The dose response multicentre investigation on fluid assessment (DoReMIFA) in critically ill patients. Crit Care. 2016;20:196. 27334608 10.1186/s13054-016-1355-9 PMC4918119

[16] Renal T . An observational study fluid balance and patient outcomes in the randomized evaluation of normal vs. augmented level of replacement therapy trial*. Crit Care Med. 2012;40:1753‐1760. 22610181 10.1097/CCM.0b013e318246b9c6

[17] Chan A‐W , Tetzlaff JM , Altman DG , et al. SPIRIT 2013 statement: defining standard protocol items for clinical trials. Ann Intern Med. 2013;158:200‐207. 23295957 10.7326/0003-4819-158-3-201302050-00583 PMC5114123

[18] Singer M , Deutschman CS , Seymour CW , et al. The Third International Consensus Definitions for Sepsis and Septic Shock (Sepsis‐3). JAMA. 2016;315:801‐810. 26903338 10.1001/jama.2016.0287 PMC4968574

[19] Poukkanen M , Wilkman E , Vaara ST , et al. Hemodynamic variables and progression of acute kidney injury in critically ill patients with severe sepsis: data from the prospective observational FINNAKI study. Crit Care. 2013;17:R295. 24330815 10.1186/cc13161 PMC4056430

[20] Silbert BI , Ho KM , Lipman J , et al. Determinants of urinary output response to IV furosemide in acute kidney injury: a pharmacokinetic/pharmacodynamic study. Crit Care Med. 2016;44(10):e923‐e929. 27183025 10.1097/CCM.0000000000001823

[21] Glassford NJ , Mårtensson J , Eastwood GM , et al. Defining the characteristics and expectations of fluid bolus therapy: a worldwide perspective. J Crit Care. 2016;35:126‐132. 27481747 10.1016/j.jcrc.2016.05.017

[22] Ukor IF , Hilton AK , Bailey MJ , Bellomo R . The haemodynamic effects of bolus versus slower infusion of intravenous crystalloid in healthy volunteers. J Crit Care. 2017;41:254‐259. 28599199 10.1016/j.jcrc.2017.05.036

[23] Bihari S , Wiersema UF , Schembri D , et al. Bolus intravenous 0.9% saline, but not 4% albumin or 5% glucose, causes interstitial pulmonary edema in healthy subjects. J Appl Physiol. 1985;2015(119):783‐792. 10.1152/japplphysiol.00356.2015 26228998

[24] Chawla LS , Davison DL , Brasha‐Mitchell E , et al. Development and standardization of a furosemide stress test to predict the severity of acute kidney injury. Crit Care. 2013;17:R207. 24053972 10.1186/cc13015 PMC4057505

[25] Kahan BC , Morris TP . Reporting and analysis of trials using stratified randomisation in leading medical journals: review and reanalysis. BMJ. 2012;345:e5840. 22983531 10.1136/bmj.e5840 PMC3444136

[26] Gomez H , Ince C , De Backer D , et al. A unified theory of sepsis‐induced acute kidney injury: inflammation, microcirculatory dysfunction, bioenergetics, and the tubular cell adaptation to injury. Shock. 2014;41:3‐11. 10.1097/SHK.0000000000000052 PMC3918942 24346647

[27] Schulz KF , Altman DG , Moher D . CONSORT 2010 statement: updated guidelines for reporting parallel group randomized trials. Ann Intern Med. 2010;152:726‐732. 20335313 10.7326/0003-4819-152-11-201006010-00232

[28] Meyhoff TS , Hjortrup PB , Møller MH , et al. Conservative vs liberal fluid therapy in septic shock (CLASSIC) trial‐protocol and statistical analysis plan. Acta Anaesthesiol Scand. 2019;63:1262‐1271. 31276193 10.1111/aas.13434

[29] Self WH , Semler MW , Bellomo R , et al. Liberal versus restrictive intravenous fluid therapy for early septic shock: rationale for a randomized trial. Ann Emerg Med. 2018;72:457‐466. 29753517 10.1016/j.annemergmed.2018.03.039 PMC6380679

[30] Prowle JR , Kirwan CJ , Bellomo R . Fluid management for the prevention and attenuation of acute kidney injury. Nat Rev Nephrol. 2014;10:37‐47. 24217464 10.1038/nrneph.2013.232

[31] Ostermann M , Liu K , Kashani K . Fluid management in acute kidney injury. Chest. 2019;156:594‐603. 31002784 10.1016/j.chest.2019.04.004

