# Supplementary material for: Noninterventional follow‐up vs fluid bolus in RESPONSE to oliguria—The RESPONSE trial protocol and statistical analysis plan
Source: Acta Anaesthesiol Scand. 2020 Apr 28;64(8):1210–7. doi: 10.1111/aas.13599 (PMC7496618; doi:10.1111/aas.13599)
Supplement: Supplementary file 1 — Supplementary Material [file AAS-64-1210-s001.pdf]

Non-interventional follow-up versus fluid bolus in RESPONSE to oliguria– The RESPONSE trial protocol and statistical analysis plan

Supplementary appendix

Index

DETAILED INCLUSION AND EXCLUSION CRITERIA 2
INCLUSION CRITERIA 2
EXCLUSION CRITERIA 2
COLLECTED DATA 4
1.BASELINE VARIABLES 4
2. VARIABLES AT RANDOMIZATION 4
3. DURING STUDY PERIOD AND UNTIL 6 HOURS POST-RANDOMIZATION 5
4. OUTCOME VARIABLES 5

Detailed inclusion and exclusion criteria

Inclusion criteria (all must be fulfilled)

- Age over 18
  - on the randomization day
- Emergency admission to an ICU
  - elective admissions (for example after elective surgery) are not eligible
- Mean arterial pressure (MAP) >65 mmHg (with vasopressors if needed) and initial fluid resuscitation (over 20ml/kg iv-fluids during the last 12hrs) for shock/hypovolemia has been given OR patient has been in the ICU over 6hrs
  - Iv-fluids includes crystalloids and blood products, but not carrier fluids for medication
- Oliguria (urine output less than 0.5mL/kg/h) for at least 2 consecutive hours
  - Patient body weight is that registered on ICU admission

Exclusion criteria (none cannot be present)

- Marked fluctuations in hemodynamics within the last 2 hours pre-randomization (cardiac arrhythmias affecting blood pressure, increase in norepinephrine need over 0.2 ug/kg/min, need for initiation of inotrope/inodilator)
- Administration of furosemide within last 6 hours
- Chronic kidney disease (estimated pre-critical illness GFR < 60ml/min/1.73m2)
- Renal replacement therapy (RRT)
  - RRT has been already started in the ICU for AKI
  - Commencing RRT (according to last laboratory values) is likely within the next 6hrs
  - Patient undergoes regular (chronic) dialyses
  - Patient has a history of kidney transplantation
- Urgent indications for commencing RRT for AKI are present (based on last blood work)
  - plasma potassium > 6mmol/l

    - severe metabolic acidosis (pH<7.20 and bicarbonate <12mmol/l),
    - evidence of severe respiratory failure (PaO2/FiO2 ratio <200) and clinical perception of volume overload
    - AKI has continued over 72hrs (creatinine remains more than twice the normal level/oliguria continues)
- Fluid overload (cumulative fluid accumulation exceeds 10% of baseline body weight)
    - Even if fluid overload has no impact on oxygenation
- Pulmonary edema (bilateral infiltrates in chest x-ray)
- Active bleeding (need for transfusion, platelets, or fresh frozen plasma)
    - operational definition: transfusion is planned within next 6 hrs
- Suspected or known intra-abdominal hypertension (intra-abdominal pressure >16mmHg)
- Pregnant or lactating
    - clinical team being aware of pregnancy/lactation
- Expected survival less than 24h
    - Patients whose ICU treatment is withdrawn
    - Strong suspicion that patient will not survive over 24hrs
    - Organ donors
- Obtaining informed written consent is not possible / consent is denied

Collected data

1.Baseline variables

Date of enrollment

Sex

Age at randomization

Measured height and weight at ICU admission

Presence of co-morbidities as recorded in the medical records: hypertension, chronic heart failure, coronary artery disease, chronic obstructive pulmonary disease, chronic liver failure, diabetes, malignancy, rheumatoid diseases

Operative or non-operative admission, type and date of surgery if operative

ICU admission diagnosis

Baseline plasma creatinine (the last value obtained 7-365 days before hospital admission if available, otherwise the lowest value during current hospitalization)

Simplified Acute Physiology Score (SAPS) II at 24h

2. Variables at randomization

Presence of AKI according to KDIGO criteria[5]

Presence on sepsis according to sepsis-3 definition[28]

Plasma creatinine

Sequential Organ Failure Assesment (SOFA) score on study enrollment day

Ventilator treatment or non-invasive treatment

Volume of fluids given, cumulative balance from ICU admission

Duration of oliguria (hours) preceding randomization

Use of corticosteroids, anticoagulation

Doses of all vasoactive drugs

Vital signs including heart rate and rhythm, arterial blood pressure, SpO_2, FiO_2, respiratory rate, central venous pressure (if measured)

Results of blood-gas analysis

Peripheral temperature and capillary refill time if available

Other existing indications for fluid bolus

3. During study period and until 6 hours post-randomization

Hourly urine output

Vital signs including heart rate and rhythm, arterial blood pressure, SpO_2, respiratory rate, central venous pressure, FiO_2 (every 15 minutes for first two hours, then every 30 minutes)

Administered vasoactive drugs (every 15 minutes for first two hours, then every 30 minutes)

Blood gas analysis at 1, 2, 4 and 6 hours

Administered other fluid therapy (hourly)

Administered diuretics

Peripheral temperature and capillary refill time if available

4. Outcome variables

Number of patients receiving rescue boluses

Serious adverse events on study day

Duration of consecutive oliguria (urine output <0.5mL/kg)

Highest AKI stage on study day, 48 hours, and during ICU stay

Number of patients receiving renal replacement therapy

Length of ICU stay

ICU and hospital mortality truncated at 30 days
